# Non-Markovianity of a Central Spin Interacting with a Lipkin–Meshkov–Glick Bath via a Conditional Past–Future Correlation

**DOI:** 10.3390/e22080895

**Published:** 2020-08-15

**Authors:** Liping Han, Jian Zou, Hai Li, Bin Shao

**Affiliations:** 1 School of Physics, Beijing Institute of Technology, Beijing 100081, China; hanlp9902@bit.edu.cn (L.H.); sbin610@bit.edu.cn (B.S.); 2 School of Science, Tianjin University of Technology, Tianjin 300384, China; 3 School of Information and Electronic Engineering, Shandong Technology and Business University, Yantai 264005, China; lihai@sdtbu.edu.cn

**Keywords:** non-Markovianity, Lipkin–Meshkov–Glick (LMG) model, conditional past–future (CPF) correlation

## Abstract

Based on conditional past–future (CPF) correlations, we study the non-Markovianity of a central spin coupled to an isotropic Lipkin–Meshkov–Glick (LMG) bath. Although the dynamics of a system is always non-Markovian, it is found that some measurement time intervals considering a specific process, with respect to a particular set of CPF measurement operators, can be zero, which means that in this case the non-Markovianity of the system could not be detected. Furthermore, the initial system–bath correlations only slightly influence the non-Markovianity of the system in our model. Significantly, it is also found that the dynamics of the system for LMG baths, initially in the ground states corresponding to the symmetric phase and symmetry broken phase, exhibit different properties, and the maximal value of the CPF at the critical point is the smallest, independent of the measurement operator, which means that the criticality can manifest itself by the CPF. Moreover, the effect of bath temperature on the quantum criticality of the CPF depends on the measurement operator.

## 1. Introduction

As is known, realistic quantum systems inevitably interact with their surrounding environments, and the dynamics of such open quantum systems [1,2,3,4,5] have attracted a lot of attention. The time evolution of open quantum systems is usually characterized by a quantum master equation through Markovian approximations [6], where the environment is assumed to be memoryless leading to a monotonic information flow from the system of interest to the environment. However, the Markovian approximation or master equation breaks down in systems with a strong coupling to their environments and structured environment spectral densities. In these cases, usually the environment exhibits memory effects, and so a backflow of information from the environment to the system occurs, namely, the system experiences non-Markovian dynamics [7,8]. Researchers have found that many relevant physical systems could not be described simply by Markovian dynamics, such as quantum dots [9], quantum optical systems [10,11], color-core spin in semiconductors [12], as well as biological systems [13], and quantum chemistry [14].

In recent years, more and more attention has been paid to the study of non-Markovian processes of open quantum systems [15,16,17,18,19,20,21,22,23,24,25,26,27,28,29,30,31,32,33,34,35,36,37,38,39,40,41,42,43,44]. Several review articles on quantum non-Markovianity are available [7,8,45]. The measure of the non-Markovianity of quantum evolution is a fundamental problem which aims to detect whether a quantum process is non-Markovian and how much it deviates from a Markovian one. Various definitions and measures of non-Markovianity have been introduced based on different physical concepts. Most of these definitions and measures for non-Markovianity were based on the following two main ideas: completely positive (CP) divisibility and information back flow. Based on CP divisibility, several measures of non-Markovianity have been proposed in [16,17,18,19,20,21]. Following the idea of information back flow, different measures were considered, such as measures based on the distinguishability of states in [22], on quantum entanglement in [16], on the quantum Fisher information flow in [23,24], on the fidelity in [25], on the mutual information in [26,27], on the channel capacity in [28], on the geometry of a set of accessible states in [29], on the channel distinguishability in [30], and on the two-time correlation functions in [31]. It is worthwhile to notice that different measures of non-Markovianity agree neither on the degree of non-Markovianity of a given process nor even on whether it is Markovian [27,32].

In fact, memory effects developed in open quantum systems can also be defined by alternative methods. For example, the well-established notion of classical Markovianity [46] can be extended to a quantum regime by subjecting the system to extra control operations (measurements). Recently, an operational definition of quantum Markovianity based on a “process tensor” framework was introduced in [41,42], which relies on the usual definition of classical Markovianity in terms of conditional probability distributions [46]. The main theoretical component of this definition is the statistical independence of past and future events when conditioned on a given state at the present time [47]. Then, Budini extended this formulation of classical Markovianity to quantum regime and proposed an operational definition of quantum non-Markovianity, which is based on a minimal set of three time-ordered successive measurements performed solely on the quantum system [43,44]. Hence, conditional past–future (CPF) independence indicates a Markovian regime, while memory effects will break CPF independence, and a hierarchical set of CPF correlations arises in non-Markovian regime. Its definition involves both predictive and retrodicted quantum probabilities [48,49]. It should be noted that the experimental implementation has been recently realized [50,51].

In [43], the authors considered a central spin model and a spin in the classical noise and studied their non-Markovian dynamics based on CPF correlations. The bath in the latter was approximated as a big qubit. In general, it is very difficult to consider the change of the bath state after a measurement on the system, which strongly influences the subsequent dynamics of the system. So the quantum regression theorem was often used which approximately supposes the bath state being unchanged [52], while in a non-Markovian regime for CPF correlations, the change of the bath state after measurement could not be ignored, leading to a violation of the quantum regression theorem. Because the change of the bath state is a major factor influencing the non-Markovianity, an exactly solvable model will be significant. As is known, interacting many-body spin environments are the representative non-Markovian environments which present many interesting non-Markovian behaviors [39,40,53,54,55,56]. The Lipkin–Meshkov–Glick (LMG) model [57], which is exactly solvable, consists of *N* spins distributed in two degenerate levels and each spin interacts with others. This model, which was first introduced to describe the tunneling of bosons between two degenerate levels in nuclei, has been proven to have a quantum phase transition (QPT) [58,59,60]. By adjusting the coupling constant, the LMG model will show two different phases, i.e., a symmetric phase and symmetry broken phase. Thus, the ground states corresponding to the two phases exhibit different properties. In [40], the authors have studied the non-Markovianity of a central spin coupled to a LMG model in the case of the bath initially being in the ground state by the trace distance definition of non-Markovianity. They found that the non-Markovianity is stronger for the bath in the symmetric phase than that in the symmetry broken phase. In fact, the trace distance between quantum states has been extensively applied to investigate non-Markovianity, which is not considered for a particular physical process, but the CPF correlation is. In this paper, based on a CPF correlation, we will study the non-Markovianity of a central spin coupled to the LMG bath, especially the effects of different particular physical processes, different initial system–bath correlations and the states of the bath on the non-Markovian dynamics. Firstly, we discuss the effect of the measurement operator on the non-Markovianity of the system. It is found that the non-Markovianity of the system could not be detected by a particular measurement operator for some measurement time intervals. In a quantum regime, the system dynamics are Markovian if the CPF correlation vanishes for arbitrary measurement operators. Equivalently, the system dynamics are non-Markovian if there exists at least one set of measurement operators such that the CPF correlation does not vanishes. It is noted that even for a non-Markovian process the value of the CPF may or may not be null for different measurement operators and different measurement time intervals. Then, we consider the role of the initial system–bath correlations on the non-Markovianity. Three types of initial system–bath correlations are considered: the first has a quantum correlation, the second has a classical correlation, and the third has no correlation. It is shown that initial system–bath correlations only slightly influence the non-Markovianity of the system in our model. At last, we discuss how the state of the bath influences the non-Markovianity of the system. It is found that with LMG being initially prepared in the ground state, the behaviors of the CPF are quite different for the two different phases, and a notable discontinuity of the sudden transition for the CPF occurs at the QPT point. In the symmetric broken phase, the maximal magnitude of the CPF increases with the increase in the coupling strength between the spins in the bath, while in the symmetric phase, it decreases with the increase in the coupling strength between the spins in the bath. At the QPT point, the maximal magnitude of the CPF is the minimum. The non-Markovianity shows critical characteristics near the QPT point in the LMG bath, which is independent of the measurement operator, and thus, this quantum criticality can manifest itself by the CPF. Moreover, the initial bath state has a significant influence on the dynamics of the system, and the effect of the bath temperature on the critical characteristics of the CPF depends on the measurement operator. For some particular measurements, although the bath slightly deviates from zero (in terms of temperature), the critical behavior of the CPF will be broken, while for other measurement operators, a higher temperature can be maintained before being destroyed.

This paper is organized as follows: In Section 2, we first introduce the physical model and three types of initial system–bath correlations. Then, we briefly review the operational definition of quantum non-Markovianity based on the CPF correlation. In Section 3, we study the dynamics of the system for different particular processes and initial system–bath correlations through the CPF correlation, as well as the effects the different states of the bath has on the dynamics of the system. Section 4 is the conclusion.

## 2. Model and Methods

### 2.1. Model

We consider a central spin-$\frac{1}{2}$ system coupled to the isotropic LMG bath and the total Hamiltonian can be written as

(1) $$H=H_{S}+H_{B}+H_{SB}$$

with

(2) $$H_{S}=-\sigma_{z}\;H_{B}=-\frac{\lambda }{N}\sum_{i<j}^{N}\left(\sigma_{x}^{i}\sigma_{x}^{j}+\sigma_{y}^{i}\sigma_{y}^{j}\right)-\sum_{i=1}^{N}\sigma_{z}^{i}\;H_{SB}=-\lambda^{'}\sum_{i=1}^{N}(\sigma_{x}^{i}\sigma_{x}+\sigma_{y}^{i}\sigma_{y})$$

Here, $H_{S}$ is the Hamiltonian of the central spin and $H_{B}$ is the Hamiltonian of the isotropic LMG bath, with λ, *N* denoting the coupling strength between the spins and the number of spins in the bath. $H_{SB}$ represents the interacting Hamiltonian between the central spin and its surrounding bath, with $\lambda^{'}$ being their coupling strength. $\sigma_{k}$ and $\sigma_{k}^{i}$, k=x,y,z(i=1,2,……N) are the Pauli matrices of the central spin and the *i*-th spin in the bath, respectively.

Since the N spins in the LMG bath are identical and indistinguishable, the dynamics of the bath can be described by collective operators as

(3) $$J_{\pm }=\sum_{i=1}^{N}\sigma_{\pm }^{i},\;J_{z}=\frac{1}{2}\sum_{i=1}^{N}\sigma_{z}^{i}$$

where $J_{z}$ and $J_{\pm }$ are the *z* component of the total spin operator and the ladder operators of the LMG bath. By means of the ladder operators of the LMG bath and the central spin, we can rewrite the total Hamiltonian as

(4) $$H=-\frac{\lambda }{N}[J_{+}J_{-}+J_{-}J_{+}-N]-2J_{z}-2\lambda^{'}[s_{+}J_{-}+s_{-}J_{+}]-2s_{z}$$

where $s_{z}=\frac{1}{2}\sigma_{z}$ and $s_{\pm }=s_{x}\pm is_{y}$ are the spin operator and the ladder operator of the central spin, respectively. 

Any N spin-$\frac{1}{2}$state invariant by atom permutation is described by the Dicke state |J,M〉 with $J=\frac{N}{2}$, where |J,M〉 is obtained by the repeated action of the symmetrical collective deexcitation operator $J_{-}$ on state |↑,↑……↑〉:

(5) $$|J,M\rangle =\sqrt{\frac{(J+M)!}{N!(J-M)!}}J_{-}^{J-M}|↑,↑\ldots \ldots ↑\rangle$$

In Equation (5), M=−J,−J+1,……J−1,J, and |↑,↑……↑〉 are the full polarized states with all spins up. |J,M〉 represents the state in which the J+M spins are in the upper level |↑〉, and J−M in the lower level |↓〉. |J,M〉 is the eigenstate of $J^{2}$ and $J_{z}$ with the corresponding eigenvalues $\frac{N}{2}(\frac{N}{2}+1)$ and M, respectively. Hence, we can choose |J,M〉 as the basis vector, and the Hamiltonian of the LMG bath would be expressed as a diagonal matrix in the Dicke representation. For convenience, we write |M〉 instead of |J,M〉 for $J=\frac{N}{2}$ in the following. In terms of the LMG model, λ=1 is the QPT point. When 0<λ<1, the bath is in the symmetry broken phase, and the ground state is $|G\rangle =|\frac{N}{2}\rangle$. When λ>1, the bath is in the symmetry phase, and the ground state is |G〉=|M〉, where M is an integer nearest to N/2λ.

By means of an invariant subspace, $H_{M}$ of H spanned by the basis vectors {|M〉⊗|↑〉, |M+1〉⊗|↓〉} (|↑〉, |↓〉 are the eigenstates of $H_{S}$), the total Hamiltonian can be expressed as a quasidiagonal matrix with the diagonal blocks:

(6) $$H_{M}=\left[\begin{array}{cc} \varepsilon & \xi \\ \xi & \chi \end{array}\right]$$

where $\varepsilon =-\frac{\lambda }{2N}[N^{2}-4M^{2}]-2M-1$, $\chi =-\frac{\lambda }{2N}[N^{2}-4{\left(M+1\right)}^{2}]-2(M+1)+1$, $\xi =-\lambda^{'}\sqrt{N(N+2)-4M(M+1)}$. Through a straightforward calculation, the system–bath unitary time evolution operator U(t)=exp(−iHt) can also be expressed in the invariant subspace $H_{M}$ of H as

(7) $$U_{M}(t)=\left[\begin{array}{cc} g^{2}e^{-ix_{1}t}+h^{2}e^{-ix_{2}t} & gh(e^{-ix_{1}t}-e^{-ix_{2}t})\\ gh(e^{-ix_{1}t}-e^{-ix_{2}t}) & h^{2}e^{-ix_{1}t}+g^{2}e^{-ix_{2}t} \end{array}\right]$$

where $x_{1}$ and $x_{2}$ are the eigenvalues of $H_{M}$, written as

$$x_{1}=\frac{1}{2}\left[(\varepsilon +\chi )+\sqrt{{\left(\varepsilon -\chi \right)}^{2}+4\xi^{2}}\right],\;x_{2}=\frac{1}{2}\left[(\varepsilon +\chi )-\sqrt{{\left(\varepsilon -\chi \right)}^{2}+4\xi^{2}}\right].$$

In Equation (7), $g=\frac{\xi }{\sqrt{{\left(\varepsilon -x_{1}\right)}^{2}+\xi^{2}}}$, $h=\frac{x_{1}-\varepsilon }{\sqrt{{\left(\varepsilon -x_{1}\right)}^{2}+\xi^{2}}}$. It is noticed that the above equation is valid only when $-\frac{N}{2}\leq M<\frac{N}{2}$. $M=\frac{N}{2}$, $|\frac{N}{2}\rangle ⊗|↑\rangle$ is an eigenstate of the total Hamiltonian which is a one-dimensional invariant subspace, and its corresponding eigenenergy is −(N+1). $|-\frac{N}{2}\rangle ⊗|↓\rangle$ is also an eigenstate of the total Hamiltonian, and its eigenenergy is (N+1).

Now, assuming the initial system–bath state is $\rho_{tot}(0)$, we can obtain the time evolution of the density matrix for the composite system by solving the following Liouville equation,

(8) $$\rho_{tot}(t)=U(t)\rho_{tot}(0)U^{†}(t)$$

and the reduced density matrix of system can be obtained by tracing over the bath degrees of freedom, $\rho_{s}(t){=\mathrm{Tr}}_{B}[\rho_{tot}(t)]$.

In general, system–bath initial correlations can play a significant role in the dynamics of quantum systems. In order to investigate the effects of different initial system–bath correlations on the dynamics of central spin, in this paper we will consider the following three types of initial states for the composite system,

$$\rho_{tot}^{1}(0)=(\alpha |\mu \rangle |0\rangle +\beta |\nu \rangle |\frac{N}{2}\rangle )\times (\alpha^{*}\langle \mu |\langle 0|+\beta^{*}\langle \nu |\langle \frac{N}{2}|)$$

$$\rho_{B}(0)⊗(\sin^{2}\gamma |↑\rangle \langle ↑|+\cos^{2}\gamma |↓\rangle \langle ↓|)$$

$$-P_{0}\cos^{2}\gamma |0↓\rangle \langle 0↓|-P_{0}sin^{2}\gamma |0↑\rangle \langle 0↑|$$

(9) $$-P_{N/2}\sin^{2}\gamma |\frac{N}{2}↓\rangle \langle \frac{N}{2}↓|-P_{N/2}\cos^{2}\gamma |\frac{N}{2}↑\rangle \langle \frac{N}{2}↑|$$

$$\rho_{tot}^{2}(0)={\left|\alpha \right|}^{2}|\mu \rangle \langle \mu |⊗|0\rangle \langle 0|+{\left|\beta \right|}^{2}|\nu \rangle \langle \nu |⊗|\frac{N}{2}\rangle \langle \frac{N}{2}|$$

$$+\rho_{B}(0)⊗(\sin^{2}\gamma |↑\rangle \langle ↑|+\cos^{2}\gamma |↓\rangle \langle ↓|)$$

$$-P_{0}\cos^{2}\gamma |0↓\rangle \langle 0↓|-P_{0}\sin^{2}\gamma |0↑\rangle \langle 0↑|$$

(10) $$-P_{N/2}\sin^{2}\gamma |\frac{N}{2}↓\rangle \langle \frac{N}{2}↓|-P_{N/2}\cos^{2}\gamma |\frac{N}{2}↑\rangle \langle \frac{N}{2}↑|$$

(11) $$\rho_{tot}^{3}(0)=\rho_{S}(0)⊗\rho_{B}(0)$$

where |μ〉=cosγ|↓〉+sinγ|↑〉, |ν〉=sinγ|↓〉−cosγ|↑〉 (γ∈[0,π]). $\rho_{S}(0)$ and $\rho_{B}(0)$ are given by

ρS(0)=cos2γ|↓〉〈↓|+(P0−PN2)cosγsinγ|↓〉〈↑|

(12) +(P0−PN2)cosγsinγ|↑〉〈↓|+sin2γ|↑〉〈↑|

(13) ρB(0)=∑M=−N2N2PM|M〉〈M|

In Equations (9)–(13), $\rho_{B}(0)$ is chosen to be a thermal state with $P_{M}=e^{-E_{M}/T_{B}}/Z$. Here, $Z=\sum_{M}e^{-E_{M}/T_{B}}$ is the partition function, $E_{M}$ is the eigenenergy of $H_{B}$ corresponding to the eigenstate |M〉, and $T_{B}$ is the bath temperature. $P_{0}$ and PN2 are the populations of the states |0〉 and $|\frac{N}{2}\rangle$, respectively. The initial states $\rho_{tot}^{1}(0)$, $\rho_{tot}^{2}(0)$ and $\rho_{tot}^{3}(0)$ have a quantum correlation, classical correlation, and no correlation, respectively. It is noticed that since our focus is on the correlations in the composite system, we set ${\left|\alpha \right|}^{2}=P_{0}$ and |β|2=PN2, and thus the three initial states will have the same reduced density matrices for both the system and the bath, i.e., Equations (12) and (13). For simplicity, N is supposed to be an even number.

### 2.2. Methods

In this section, we will review the CPF definition of quantum non-Markovianity based on a minimal set of three time-ordered successive measurements in [43]. Considering an observation of a classical stochastic system at three successive times $t_{x}<t_{y}<t_{z}$, with the outcomes of x, y, z, the Bayes rule allows us to write the conditional probability P(z,x|y) of past (x) and future events (z) given the present state (y), as

(14) P(z,x|y)=P(z|y,x)P(x|y)

where, in general, P(x|y) stands for the conditional probability of x given y. For a classical Markovian process, from the fact that past and future events become statistically independent when conditioned on a given intermediate state, we can obtain P(z,x|y)=P(z|y)P(x|y). This property can be corroborated with the CPF, which is defined as

(15) $$C_{pf}={\langle O_{z}O_{x}\rangle }_{y}-{\langle O_{z}\rangle }_{y}{\langle O_{x}\rangle }_{y}$$

where O corresponds to a property or quantity related to each system state. $C_{pf}$ can be expressed in the form of probability distributions as

(16) $$C_{pf}=\sum_{zx}\left[P(z,x|y)-P(z|y)P(x|y)\right]O_{z}O_{x}$$

where the sum indexes z and x run over all possible outcomes occurring at times $t_{z}$ and $t_{x}$, respectively, while y is a fixed particular possible value at time $t_{y}$. The property of P(z,x|y)=P(z|y)P(x|y) for a Markovian process leads to $C_{pf}=0$, whatever the conditional state y is. In contrast, for non-Markovian process, it is expected that $C_{pf}\neq 0$. 

The definition of $C_{pf}$ mentioned above can also be extended to a quantum regime, where the sequence x, y, z is altered by the outcomes of three successive quantum measurements performed on the system of interest. The corresponding measurement operators are $\Omega_{x}$, $\Omega_{y}$, $\Omega_{z}$, and satisfy $\sum_{x}\Omega_{x}^{†}\Omega_{x}$= $\sum_{y}\Omega_{y}^{†}\Omega_{y}$= $\sum_{z}\Omega_{z}^{†}\Omega_{z}=I$, where I is the identity matrix in the system Hilbert space and the sum indexes run over all possible outcomes at each stage. It is noticed that given x in the past of y, P(x|y) is a retrodicted probability which can be read from a “past quantum state ” formalism. In a quantum regime, the system dynamics are Markovian if, for arbitrary measurement operators, the CPF correlation vanishes, i.e., $C_{pf}=0$. Equivalently, the system dynamics are non-Markovian if there exists at least one set of measurement operators such that the CPF correlation does not vanish, i.e., $C_{pf}\neq 0$ [50]. Hence, this property of $C_{pf}$ guarantees its application as a measure of non-Markovianity.

With the help of $C_{pf}$, we investigate the non-Markovianity of a central spin with LMG bath. In this paper, the three measurement operators are chosen as the arbitrary projective ones, and all of them are the same, which can be expressed through the Bloch vectors, as $\Omega_{\pm }=|\psi_{\pm }\rangle \langle \psi_{\pm }|$, with $|\psi_{+}\rangle =\cos\frac{\theta }{2}|↑\rangle +e^{i\varphi }\sin\frac{\theta }{2}|↓\rangle$ and $|\psi_{-}\rangle =\sin\frac{\theta }{2}|↑\rangle -e^{i\varphi }\cos\frac{\theta }{2}|↓\rangle$. Here, θ∈[0,π] and φ∈[0,2π]. Firstly, we perform the first measurement $\Omega_{x}$ on the central spin at $t_{x}=0$. After the measurement, the density matrix of the composite system suffers a transformation $\rho (0)\to \rho^{x}(0)=\frac{\Omega_{x}\rho (0)\Omega_{x}^{†}}{tr[\Omega_{x}\Omega_{x}^{†}\rho (0)]}$, where x=±1 is the outcome of the measurement. The probabilities of both outcomes are $P(x=1)=tr[\Omega_{+}\Omega_{+}^{†}\rho (0)]$ and $P(x=-1)=tr[\Omega_{-}\Omega_{-}^{†}\rho (0)]$, respectively. In the next step, during a time interval $t=t_{y}-t_{x}$ (after the first measurement and before the second measurement), the composite system evolves unitarily as

(17) $$\rho^{x}(0)\to \rho^{x}(t)=U(t)\rho^{x}(0)U^{†}(t)$$

Next, the second measurement $M_{y}$ with outcomes being y=±1, is performed at time $t_{y}$. The whole composite system state changes as $\rho^{x}(t)\to \rho^{yx}(t)=\frac{\Omega_{y}\rho^{x}(t)\Omega_{y}^{†}}{tr[\Omega_{y}\Omega_{y}^{†}\rho^{x}(t)]}$ after the second measurement. Given that the previous outcome was x, the probability of each result is $P(y|x)=tr[\Omega_{y}\Omega_{y}^{†}\rho^{x}(t)]$. Then, the composite system evolves during a time interval $\tau =t_{z}-t_{y}$ (after the second measurement and before the last measurement) unitarily once more which can be obtained as

(18) $$\rho^{\left(yx\right)}(t)\to \rho^{\left(yx\right)}(t+\tau )=U(\tau )\rho^{\left(yx\right)}(t)U^{†}(\tau )$$

The probability of the last measurement $M_{z}$ is $P(z|y,x)=tr[\Omega_{z}\Omega_{z}^{†}\rho^{\left(yx\right)}(t+\tau )]$. From P(x), P(y|x) and P(z|y,x) mentioned above, we can obtain the probabilities P(x|y), P(z,x|y) and P(z|y), as

(19) P(x|y)=P(y,x)/P(y)

(20) P(z,x|y)=P(z|y,x)P(x|y)

(21) $$P(z|y)=\sum_{x=\pm 1}P(z,x|y)$$

where P(y,x)=P(y|x)P(x) and $P(y)=\sum_{x=\pm 1}P(y,x)$. Then, substituting Equations (19)–(21) into Equation (16), the exact expression for $C_{pf}$ can be obtained. It is noticed that we choose both measurement time-intervals t and τ to be equal in this paper.

## 3. Effects of Different Factors on $C_{pf}$

### 3.1. Effect of Measurement Operators on $C_{pf}$

**Figure 1:**
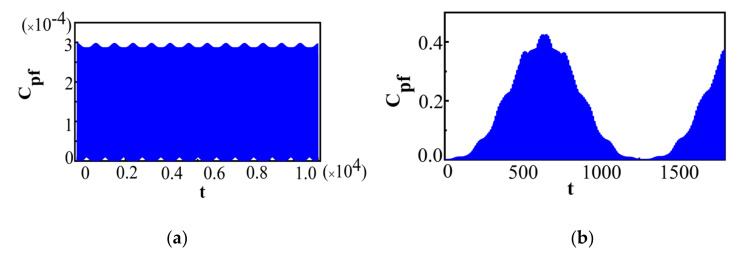
For two different measurement operators, (**a**) for $\Omega_{\hat{z}=\pm 1}$ and (**b**) for $\Omega_{\hat{x}=\pm 1}$. In both cases, the initial states are chosen to be the same as $\rho_{\mathrm{tot}}^{3}$(0) and the parameters are N = 300, γ = 0.98, $\gamma^{'}$ = 0.002, *T_B_* = 0.01, =/3, γ=π3.

As is known, the behavior of $C_{pf}$ depends on the measurement operators and the measurement time intervals, and thus $C_{pf}$ may be equal to or not equal to zero for the same dynamical process. When we perform a measurement on the system at the initial time, the system will collapse into the state corresponding to the measurement operator used. For different measurement operators, the system will collapse into different states, and thus the behavior of $C_{pf}$ will be different. To show this, we plot $C_{pf}$ for two different measurement operators in Figure 1a for the measurement in the $\hat{z}$-direction in the Bloch sphere of the qubit $\Omega_{\hat{z}=\pm 1}$ (*θ* = 0) and Figure 1b for the measurement in the $\hat{x}$- direction in the Bloch sphere of the qubit $\Omega_{\hat{z}=\pm 1}$ (*θ* = *π*/2), with the intermediate y-outcome being 1. $\Omega_{\hat{z}=\pm 1}$ performing on the system makes the system collapse into the state |↑〉 with an outcome of 1, and the state |↓〉 with an outcome of −1. $\Omega_{\hat{x}=\pm 1}$ performing on the system makes the system collapse into the state $\frac{1}{\sqrt{2}}(|↑\rangle +|↓\rangle )$ with an outcome of 1, and the state $\frac{1}{\sqrt{2}}(|↑\rangle -|↓\rangle )$ with an outcome of −1. For simplicity, we choose the initial state without any correlation, i.e., Equation (11), for both cases in Figure 1, and other parameters are chosen as N=300, λ=0.98, $\lambda^{'}=0.002$, $T_{B}=0.01$ and γ=π3. $C_{\mathrm{pf}}$ displays the periodic oscillations for both measurement operators, but the oscillatory behaviors in these two cases are obviously different.

The amplitude of $C_{pf}$ for $\Omega_{\hat{z}=\pm 1}$ remains almost unchanged which can be seen in Figure 1a, while $\Omega_{\hat{x}=\pm 1}$ exhibits periodic collapses and revivals in Figure 1b. The amplitude in Figure 1a being almost without decay means that the maximum deviation from Markovianity almost does not decrease for $\Omega_{\hat{x}=\pm 1}$, while the amplitude in Figure 1b can decay nearly to zero for quite a long time, which indicates that the non-Markovianity of the system could not be detected by the measurement operator $\Omega_{\hat{x}=\pm 1}$ for some measurement time intervals. In general, the time interval where the non-Markovianity of the system could not be detected gradually becomes longer with θ varying from 0 to π/2, while it gradually becomes shorter when θ increases from π/2 to π. When θ reaches π, the behavior of $C_{pf}$ is similar to that of $\Omega_{\hat{z}=\pm 1}$. The different behaviors of $C_{pf}$ for different measurement operators imply that $C_{pf}$ depends on the specific implementation process, and it may be equal to or not equal to zero for different measurement operators and different measurement time intervals. It is worth noting that though the dynamics of the system are non-Markovian, $C_{pf}=0$ implies that the non-Markovianity could not be detected by a particular measurement operator for some measurement time intervals, such as a particular process with respect to a measurement $\Omega_{\hat{x}=\pm 1}$.

### 3.2. Effect of the Initial Correlation on $C_{pf}$

In general, non-Markovianity is influenced by both an information backflow and the initial system–bath correlation. We will show in this subsection how the behavior of $C_{pf}$ is affected by the initial system–bath correlations. Since our focus is on the correlations in the composite system, we choose the same measurement operator for these three different initial states. Different from the part in Section 3.1, when we perform the same measurement on the system at the initial time, due to the different types of initial system–bath correlations, the bath may collapse into different states. Thus, the behavior of $C_{pf}$ will be different. After the first measurement $M_{x}$, the density matrix $\rho_{tot}^{x}(0)$ can be obtained and the system collapses into the same state corresponding to the measurement operator used. In contrast, the bath may collapse into different states due to the different initial system–bath correlations. After the first measurement $M_{x}$ with x=1, the $\rho_{B}^{x}(0)$ for three different initial system–bath correlations are, respectively,

$$\rho_{B}^{(1)x}(0)=\frac{1}{P^{1}(x=1)}\{P_{0}[cos^{2}\gamma {\left|c\right|}^{2}+cos\gamma sin\gamma (b^{*}+b)+sin^{2}\gamma {\left|a\right|}^{2}+{\left|b\right|}^{2}]|0\rangle \langle 0|$$

+PN2[cos2γ|c|2−cosγsinγ(b*+b)+sin2γ|a|2+|b|2]|N2〉〈N2|

(22) $$+[\sum_{M=-N/2}^{-1}P_{M}(sin^{2}\gamma {\left|a\right|}^{2}+cos^{2}\gamma {\left|c\right|}^{2})+\sum_{M=1}^{N/2-1}P_{M}(sin^{2}\gamma {\left|a\right|}^{2}+cos^{2}\gamma {\left|c\right|}^{2})]|M\rangle \langle M|\}$$

$$\rho_{B}^{(2)x}(0)=\frac{1}{P^{2}(x=1)}\{P_{0}[cos^{2}\gamma {\left|c\right|}^{2}+cos\gamma sin\gamma (b^{*}+b)+sin^{2}\gamma {\left|a\right|}^{2}+{\left|b\right|}^{2}]|0\rangle \langle 0|$$

+PN2[cos2γ|c|2−cosγsinγ(b*+b)+sin2γ|a|2+|b|2]|N2〉〈N2|

(23) $$+[\sum_{M=-N/2}^{-1}P_{M}(sin^{2}\gamma {\left|a\right|}^{2}+cos^{2}\gamma {\left|c\right|}^{2})+\sum_{M=1}^{N/2-1}P_{M}(sin^{2}\gamma {\left|a\right|}^{2}+cos^{2}\gamma {\left|c\right|}^{2})]|M\rangle \langle M|\}$$

(24) $$\begin{array}{l} \rho_{B}^{(3)x}(0)=\sum_{M=-N/2}^{N/2}P_{M}|M\rangle \langle M| \end{array}$$

with

$$P^{1}(x=1)=P_{0}[cos^{2}\gamma {\left|c\right|}^{2}+cos\gamma sin\gamma (b^{*}+b)+sin^{2}\gamma {\left|a\right|}^{2}+{\left|b\right|}^{2}]$$

+PN2[cos2γ|c|2−cosγsinγ(b*+b)+sin2γ|a|2+|b|2]

(25) $$+[\sum_{M=-N/2}^{-1}P_{M}(sin^{2}\gamma {\left|a\right|}^{2}+cos^{2}\gamma {\left|c\right|}^{2})+\sum_{M=1}^{N/2-1}P_{M}(sin^{2}\gamma {\left|a\right|}^{2}+cos^{2}\gamma {\left|c\right|}^{2})]$$

$$P^{2}{\left(x=1\right)}^{2}=P_{0}[cos^{2}\gamma {\left|c\right|}^{2}+cos\gamma sin\gamma (b^{*}+b)+sin^{2}\gamma {\left|a\right|}^{2}+{\left|b\right|}^{2}]$$

+PN2[cos2γ|c|2−cosγsinγ(b*+b)+sin2γ|a|2+|b|2]

(26) $$+[\sum_{M=-N/2}^{-1}P_{M}(sin^{2}\gamma {\left|a\right|}^{2}+cos^{2}\gamma {\left|c\right|}^{2})+\sum_{M=1}^{N/2-1}P_{M}(sin^{2}\gamma {\left|a\right|}^{2}+cos^{2}\gamma {\left|c\right|}^{2})]$$

**Figure 2:**
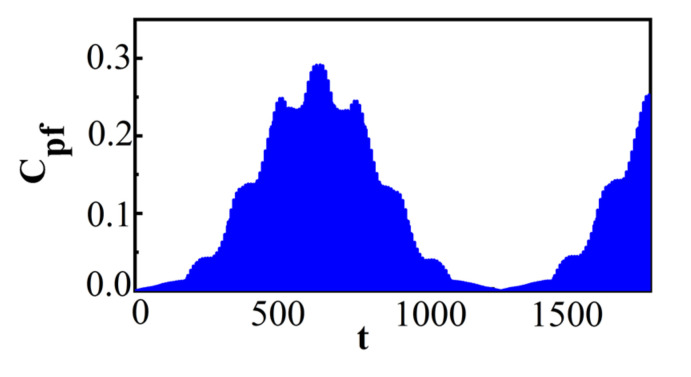
For $\rho_{tot}^{2}(0)$ with $\Omega_{\hat{x}=\pm 1}$. The parameters are the same as those in Figure 1.

$\rho_{B}^{(1)x}(0)$, $\rho_{B}^{(2)x}(0)$ and $\rho_{B}^{(3)x}(0)$ are the density matrices of the bath after the first measurement for three different initial states with a quantum correlation, classical correlation, and no correlation, respectively. It is noticed that $\rho_{B}^{(3)x}(0)$ is obviously the same as the initial bath state. $P^{1}(x=1)$ and $P^{2}(x=1)$ are the probabilities for outcome x=1 when we choose the initial states with quantum correlations and classical correlations, respectively. In Equations (22)–(26), $a=\cos^{2}\frac{\theta }{2}$, $b=e^{i\varphi }\cos\frac{\theta }{2}\sin\frac{\theta }{2}$ and $c=\sin^{2}\frac{\theta }{2}$. When we take $\Omega_{\hat{z}=\pm 1}$ as the measurement operator, the bath collapses into the same state for all these three correlations, and thus the behaviors of $C_{pf}$ for $\rho_{tot}^{\left(1\right)}(0)$ and $\rho_{tot}^{\left(2\right)}(0)$ will be the same for $\rho_{tot}^{\left(3\right)}(0)$. In contrast, for $\Omega_{\hat{x}=\pm 1}$, we find that $\rho_{B}^{(1)x}(0)$ and $\rho_{B}^{(2)x}(0)$ are the same, while $\rho_{B}^{(3)x}(0)$ is different. Thus, the behaviors of $C_{pf}$ are the same for $\rho_{tot}^{\left(1\right)}(0)$ and $\rho_{tot}^{\left(2\right)}(0)$, and Figure 2 plots $C_{pf}$ for $\rho_{tot}^{\left(2\right)}(0)$ only.

The parameters in Figure 2 are the same as those in Figure 1. The same behavior of $C_{pf}$ for $\rho_{tot}^{\left(1\right)}(0)$ and $\rho_{tot}^{\left(2\right)}(0)$ means that the maximum deviation from Markovianity in these two cases is the same. While the behaviors of $C_{pf}$ for $\rho_{tot}^{\left(1\right)}(0)$ and $\rho_{tot}^{\left(2\right)}(0)$ are slightly different from that for $\rho_{tot}^{\left(3\right)}(0)$. The maximal amplitude of $C_{pf}$ in Figure 2 is smaller than that in Figure 1b, which means that the maximum deviation from Markovianity for $\rho_{tot}^{\left(1\right)}(0)$ and $\rho_{tot}^{\left(2\right)}(0)$ (see Figure 2) is smaller than that for $\rho_{tot}^{\left(3\right)}(0)$ (see Figure 1b). Though the amplitudes of $C_{pf}$ are different, the time interval for $C_{pf}=0$ in Figure 2 is almost the same as that in Figure 1b, which indicates that the time interval where the non-Markovianity of the system could not be detected in these particular processes, with respect to a measurement $\Omega_{\hat{x}=\pm 1}$, for some measurement time intervals under three different initial correlations was similar. Overall, the behaviors of $C_{pf}$ are similar for these three types of initial states for an arbitrary measurement operator. This phenomenon can be understood as follows: the reasons behind a non-Markovian process, revealed in [54], consist of two different contributions. One is the presence and evolution of initial system–bath correlation and the other is the effect of the bath state. Moreover, it has been found that the limit t→0, $C_{pf}$ allows for the detection of initial correlations [50] in the same way the initial system–bath correlations can also be detected for our model. However, $C_{pf}$ contains three measurements, and the initial system–bath correlations werebroken at the first measurement, and thus the influence of the initial system–bath correlations on a non-Markovian process disappeared for this specific process. It implies that the correlations themselves could not affect the non-Markovianity for a specific measurement operator. However, the evolution of the bath state still relies on the initial system–bath correlation, and thus the initial system–bath correlations can have some influence on the evolution of the system state through the bath state indirectly. On the other hand, different collapsing bath states after measurements can lead to different values of $C_{pf}$. So, the initial states with different types of correlations can have some influence on $C_{pf}$ in our model. If the collapsing bath states are quite different, the influence of different types of correlations will be remarkable.

### 3.3. Effect of $\lambda^{'}$ on $C_{pf}$

**Figure 3:**
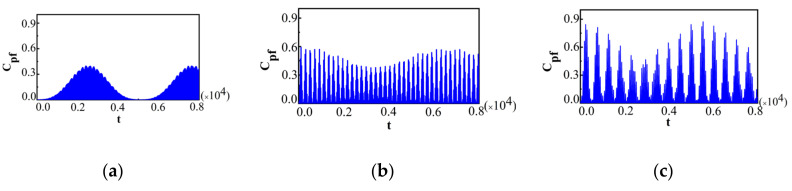
For $\Omega_{\hat{x}=\pm 1}$ and different $\lambda^{'}$; (**a**) $\lambda^{'}=0.001$; (**b**) $\lambda^{'}=0.005$ and (**c**) $\lambda^{'}=0.01$. The initial state is $\rho_{tot}^{3}(0)$ and the other parameters are the same as those in Figure 1.

In the following, we will discuss the effects of $\lambda^{'}$ on $C_{pf}$ for $\Omega_{\hat{x}=\pm 1}$ and $\Omega_{\hat{z}=\pm 1}$, respectively. Figure 3 plots $C_{pf}$ for the initial state with no correlation and different $\lambda^{'}$ with $\Omega_{\hat{x}=\pm 1}$.

**Figure 4:**
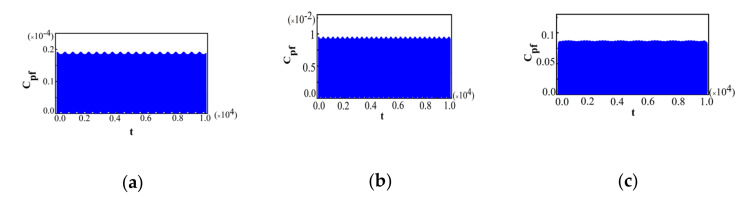
For $\Omega_{\hat{z}=\pm 1}$ and different $\lambda^{'}$; (**a**) $\lambda^{'}=0.001$; (**b**) $\lambda^{'}=0.005$and (**c**) $\lambda^{'}=0.01$. The initial state is $\rho_{tot}^{3}(0)$ and the other parameters are the same as those in Figure 1.

The value of $\lambda^{'}$ was chosen to be $\lambda^{'}=0.001$ for Figure 3a, $\lambda^{'}=0.005$ for Figure 3b, and $\lambda^{'}=0.01$ for Figure 3c, respectively, and the other parameters are the same as those in Figure 1. As shown in Figure 3, the maximal amplitude of $C_{pf}$ for $\Omega_{\hat{x}=\pm 1}$ increased with the increase in $\lambda^{'}$. It indicates that with the increase in $\lambda^{'}$, the maximum deviation from Markovianity gets larger. Moreover, the time interval where the non-Markovianity of the system could not be detected, with respect to measurement $\Omega_{\hat{x}=\pm 1}$, for some measurement time intervals is extremely sensitive to $\lambda^{'}$. With the increase in $\lambda^{'}$, this time interval became shorter and even vanished. Figure 3 demonstrates that the coupling strength $\lambda^{'}$ between the system and the bath had a remarkable influence on the time interval where the non-Markovianity of the system could not be detected with respect to a measurement $\Omega_{\hat{x}=\pm 1}$ for some measurement time intervals. Only when $\lambda^{'}$ is small enough does the time interval where the non-Markovianity of the system could not be detected become quite long. Figure 4 plots $C_{pf}$ for $\Omega_{\hat{z}=\pm 1}$, and the value of $\lambda^{'}$ as well as the other parameters shown in Figure 3, specifically, a for $\lambda^{'}=0.001$, b for $\lambda^{'}=0.005$, and c for $\lambda^{'}=0.01$.

From Figure 4, it can be seen that with the increase in $\lambda^{'}$, the maximal amplitude of $C_{pf}$ increases. This indicates that, with the increase in $\lambda^{'}$, the maximum deviation from Markovianity also gets larger for $\Omega_{\hat{z}=\pm 1}$, which is similar to that of $\Omega_{\hat{x}=\pm 1}$. It is noted that in the limit $\lambda^{'}\to 0$, the system dynamics become unitary and then $C_{pf}$ vanishes. It was expected that by increasing $\lambda^{'}$, the maximal amplitude of $C_{pf}$ would increase, and thus the maximum deviation from the Markovianity would also increase. However, $\lambda^{'}$ for $\Omega_{\hat{z}=\pm 1}$ has a greater impact on the maximal magnitude of $C_{pf}$ than it does for $\Omega_{\hat{x}=\pm 1}$. The maximal value of $C_{pf}$ for $\Omega_{\hat{z}=\pm 1}$ is in different orders of magnitude, while for $\Omega_{\hat{x}=\pm 1}$ it is in the same order of magnitude when $\lambda^{'}$ changes, which can be seen from Figure 3 and Figure 4. The maximal value of $C_{pf}$ changes more dramatically with different $\lambda^{'}$ for $\Omega_{\hat{z}=\pm 1}$ than for $\Omega_{\hat{x}=\pm 1}$.

### 3.4. Effects of the Bath on $C_{pf}$

In this subsection, we will consider the influence of the coupling strength between the spins in the bath λ and the bath temperature $T_{B}$ on the behavior of $C_{pf}$ with two different measurement operators, $\Omega_{\hat{x}=\pm 1}$ and $\Omega_{\hat{z}=\pm 1}$, respectively, in the following.

**Figure 5:**
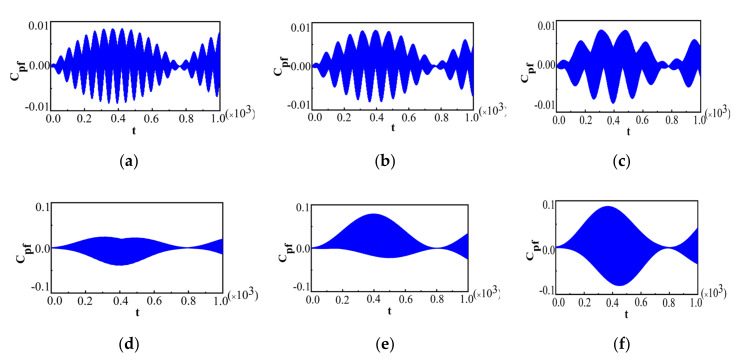
For $\Omega_{\hat{x}=\pm 1}$ and different λ near the quantum phase transition (QPT) point in the case of N=500, $\lambda^{'}=0.002$; (**a**) λ=0.97; (**b**) λ=0.98; (**c**) λ=0.99; (**d**) λ=1.01; (**e**) λ=1.02; (**f**) λ=1.03.

First of all, in order to display the effect of λ near the QPT point of the dynamics of the system, we plotted $C_{pf}$ for two different measurement operators, $\Omega_{\hat{x}=\pm 1}$ and $\Omega_{\hat{z}=\pm 1}$, in two different phases. We chose the initial system state as $\rho_{s}(0)=|↑\rangle \langle ↑|$ for $\Omega_{\hat{x}=\pm 1}$ and $\rho_{s}(0)=|+\rangle \langle +|$, with $|+\rangle =\frac{1}{\sqrt{2}}(|↑\rangle +|↓\rangle )$ for $\Omega_{\hat{z}=\pm 1}$, respectively. The initial bath state was chosen to be the ground state. We plotted $C_{pf}$ for $\Omega_{\hat{x}=\pm 1}$ and different λ in Figure 5, with λ=0.97 for Figure 5a, λ=0.98 for Figure 5b, λ=0.99 for Figure 5c, λ=1.01 for Figure 5d, λ=1.02 for Figure 5e, and λ=1.03 for Figure 5f. 

**Figure 6:**
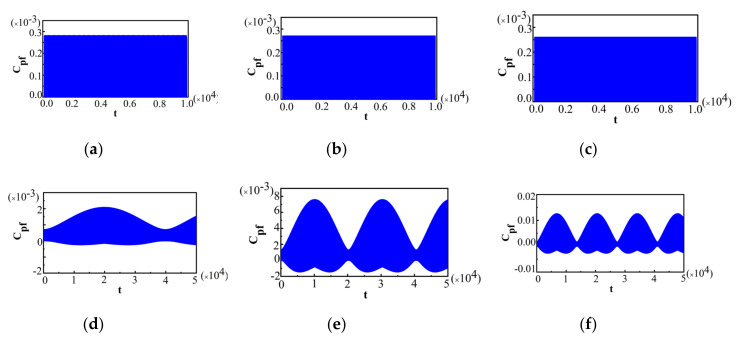
For $\Omega_{\hat{z}=\pm 1}$ and different λ near the QPT point in the case of N=500; $\lambda^{'}=0.002$; (**a**) λ=0.97; (**b**) λ=0.98; (**c**) λ=0.99; (**d**) λ=1.01; (**e**) λ=1.02; (**f**) λ=1.03.

The other parameters were chosen as N=500, $\lambda^{'}=0.002$. It was found that the behaviors of $C_{pf}$ were quite different in these two phases, and a notable discontinuity of sudden transitions for $C_{pf}$ occurred at the QPT point (λ=1). In the symmetric broken phase, we observed the phenomenon of beats as shown in Figure 5a–c. From Figure 5a–c, it can be seen that with the increase in λ, the maximal amplitude of $C_{pf}$ decreases. However, compared with Figure 5a–c, in the symmetric phase, Figure 5d–f demonstrates the periodic collapses and revivals, and that the amplitude of $C_{pf}$ can decay nearly to zero for quite a long time, which indicates that the non-Markovianity of the system could not be detected by this measurement operator $\Omega_{\hat{x}=\pm 1}$ for some measurement time intervals. This phenomenon is similar to that for $\Omega_{\hat{x}=\pm 1}$ with the initial state $\rho_{tot}^{3}(0)$ (see Figure 1b). When λ increases, the maximal amplitude of $C_{pf}$ increases. The critical behavior of $C_{pf}$ shows that with the increase in λ, the maximum magnitude of $C_{pf}$ decreased when λ<1, while the opposite situation will happen when λ>1. It implies that the maximum magnitude of $C_{pf}$ at λ=1 is the smallest. Then, we plotted $C_{pf}$ for $\Omega_{\hat{z}=\pm 1}$ and different λ in Figure 6, with a, b, and c in the symmetric broken phase, and d, e, and f in the symmetric phase. 

**Figure 7:**
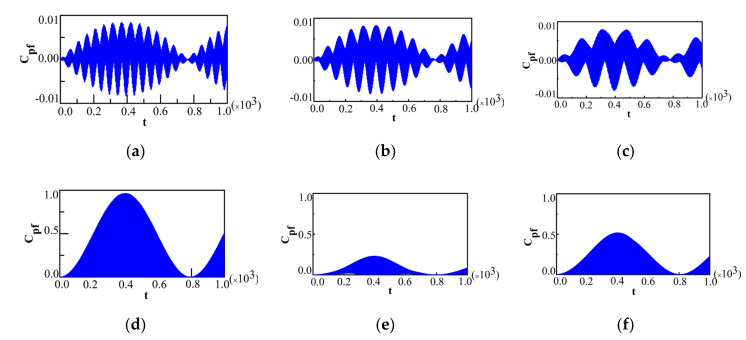
For $\Omega_{\hat{x}=\pm 1}$ and different λ near the QPT point in the case of $T_{B}=0.001$; (**a**) λ=0.97; (**b**) λ=0.98; (**c**) λ=0.99; (**d**) λ=1.01; (**e**) λ=1.02; (**f**) λ=1.03. The other parameters are the same as those in Figure 5.

**Figure 8:**
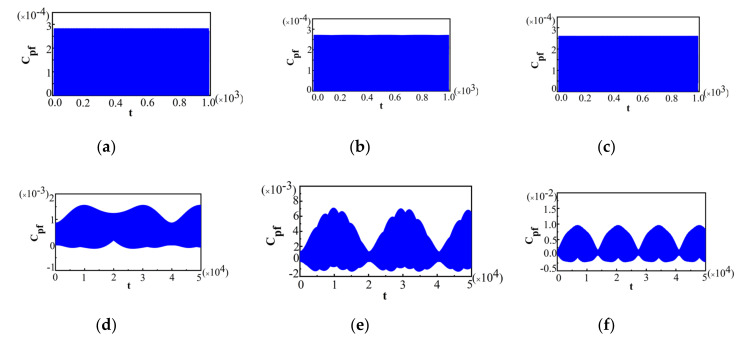
For $\Omega_{\hat{z}=\pm 1}$ and different λ near the QPT point in the case of $T_{B}=0.001$; (**a**) λ=0.97; (**b**) λ=0.98; (**c**) λ=0.99; (**d**) λ=1.01; (**e**) λ=1.02; (**f**) λ=1.03. The other parameters are the same as those in Figure 5.

The value of λ was chosen to be the same as those in Figure 5. The behaviors of $C_{pf}$ in Figure 6 are also obviously different in these two phases, and show some notable critical characteristics near the QPT point, which is similar to that for $\Omega_{\hat{x}=\pm 1}$ in Figure 5. $C_{pf}$ presents the oscillatory dynamics with equal amplitude in the symmetric broken phase as shown in Figure 6a–c, while, $C_{pf}$ in the symmetric phase, as shown in Figure 6d–f, is quite different from those in the symmetric broken phase. On the other hand, from Figure 6a–c, it can be seen that with the increase in λ, the maximal amplitude of $C_{pf}$ decreases, while, it increases (as shown in Figure 6d–f). Overall, near the QPT point, the behaviors of $C_{pf}$ shows similar critical characteristics for $\Omega_{\hat{x}=\pm 1}$ and $\Omega_{\hat{z}=\pm 1}$, and thus the critical behaviors of non-Markovianity are also similar. For other arbitrary measurement operators, with the varying of θ from 0 to π, the critical property of $C_{pf}$ is also the same as above. Therefore, there is an obvious abrupt transition for $C_{pf}$ which is independent of the measurement operator. Thus, this quantum criticality can be manifested by $C_{pf}$. Then, in order to show the effect of bath temperature $T_{B}$ on the dynamics of the system for two different measurement operators, $\Omega_{\hat{x}=\pm 1}$ and $\Omega_{\hat{z}=\pm 1}$, we choose the thermal state as the initial bath state for both cases, which is given in Equation (13). The initial system states were chosen to be the same as those for $\Omega_{\hat{x}=\pm 1}$ in Figure 5 and $\Omega_{\hat{z}=\pm 1}$ in Figure 6, respectively. We plot $C_{pf}$ for $\Omega_{\hat{x}=\pm 1}$ and $\Omega_{\hat{z}=\pm 1}$ near the QPT point for $T_{B}=0.001$in Figure 7 and Figure 8, respectively, with a, b, and c in the symmetric broken phase, and d, e, and f in the symmetric phase.

The value of λ and the other parameters were chosen to be the same as those in Figure 5. From Figure 7, we can see that although the behaviors of $C_{pf}$ on both sides of the QPT point in the LMG bath are still different, the critical characteristic of the maximal amplitude of $C_{pf}$ is broken. The maximal value of $C_{pf}$ at the QPT point is not the minimum. It means that the influence of the bath temperature is remarkable. Although the bath slightly deviates from zero temperature, the critical behavior of $C_{pf}$ for $\Omega_{\hat{x}=\pm 1}$ will be broken. Comparing to the case of $\Omega_{\hat{x}=\pm 1}$, the behaviors of $C_{pf}$ for $\Omega_{\hat{z}=\pm 1}$ in Figure 8 were also obviously different in the two different phases, and there was still a notable discontinuity of sudden transition for $C_{pf}$ at the QPT point (λ=1). The maximal value of $C_{pf}$ at the QPT point is still the minimum, which means that the critical characteristics of $C_{pf}$ shows the same result as that when $T_{B}=0$ in Figure 6. Moreover, it is found that this critical behavior of $C_{pf}$for $\Omega_{\hat{z}=\pm 1}$ can be preserved even when $T_{B}=0.01$, which is quite different from that for $\Omega_{\hat{x}=\pm 1}$ (see Figure 7). Above all, we can conclude that the effects of bath temperature $T_{B}$ on $C_{pf}$ are quite different for different measurement operators. The critical characteristics of $C_{pf}$ for $\Omega_{\hat{x}=\pm 1}$ are more sensitive to bath temperature and can be destroyed even at very low temperatures.

## 4. Conclusions

In this paper, we have studied the quantum non-Markovian dynamics of a central spin interacting with an isotropic Lipkin–Meshkov–Glick (LMG) bath through a conditional past–future (CPF) correlation. It has been found that the influence of different measurement operators on the CPF is remarkable. In particular, obvious collapses and revivals for the CPF appear when $\Omega_{\hat{x}=\pm 1}$ is performed on the system, and thus the non-Markovianity of the system could not be detected for some measurement time intervals in this particular process with respect to a measurement in the $\hat{x}$-direction in the Bloch sphere. Then, three types of initial states with different correlations between the system and the bath have been considered, i.e., a quantum correlation, classical correlation and no-correlation. However, the dynamics of the system for these three types of initial correlations are similar. It implies that the correlations themselves can have only a little influence on the CPF in our model. Although the correlations themselves could not affect the non-Markovianity, the evolution of the bath state still relies on the initial system–bath correlation, and thus the initial system–bath correlations can have some influence on the evolution of the system state through the bath state indirectly. Different collapsing bath states after measurements can lead to a different CPF. If the collapsing bath states are quite different, the influence of different types of correlations will be remarkable. Significantly, we have studied the effect of the bath on the CPF and a notable discontinuity of sudden transitions for the CPF occurs at the QPT point. In the symmetric broken phase, the maximal value of the CPF increases with the increase in λ, and in the symmetric phase, it decreases with the increase in λ. At the QPT point, the maximal value of the CPF is the minimum. The CPF shows critical characteristics near the QPT point in the LMG bath, which is independent of the measurement operator, and thus, this quantum criticality can be manifested by the CPF. Moreover, the effect of bath temperature on the critical characteristics of the CPF depends on measurement operator. The critical characteristic of the CPF is more sensitive to the bath temperature for $\Omega_{\hat{x}=\pm 1}$ than that for $\Omega_{\hat{z}=\pm 1}$. For $\Omega_{\hat{x}=\pm 1}$, although the bath slightly deviates from zero temperature, the critical behavior of the CPF will be broken, while for $\Omega_{\hat{z}=\pm 1}$ it can be maintained to a higher temperature before being destroyed.

Different from other definitions of quantum non-Markovianity, the CPF correlation provides us an operational correlation based on a minimal set of three time-ordered successive measurements performed solely on the quantum system. For the CPF correlation, the change of the bath state after a measurement generally could not be ignored, which makes it difficult to cope with. In this paper we have considered a LMG bath, and found that the CPF can witness quantum phase transitions. We expect that it can also manifest a quantum phase transition for other spin chain baths.
